# Bioactives and their roles in bone metabolism of osteoarthritis: evidence and mechanisms on gut-bone axis

**DOI:** 10.3389/fimmu.2023.1323233

**Published:** 2024-01-03

**Authors:** Sanjay Basak, Kota Sri Naga Hridayanka, Asim K. Duttaroy

**Affiliations:** ^1^ Molecular Biology Division, National Institute of Nutrition, Indian Council of Medical Research, Hyderabad, India; ^2^ Department of Nutrition, Institute of Basic Medical Sciences, Faculty of Medicine, University of Oslo, Oslo, Norway

**Keywords:** bioactive, bone remodeling, gut microbiota, inflammation, obesity, osteoarthritis, metabolism

## Abstract

Bioactives significantly modify and maintain human health. Available data suggest that Bioactives might play a beneficial role in chronic inflammatory diseases. Although promised, defining their mechanisms and opting to weigh their benefits and limitations is imperative. Detailed mechanisms by which critical Bioactives, including probiotics and prebiotics such as dietary lipids (DHA, EPA, alpha LA), vitamin D, polysaccharides (fructooligosaccharide), polyphenols (curcumin, resveratrol, and capsaicin) potentially modulate inflammation and bone metabolism is limited. Certain dietary bioactive significantly impact the gut microbiota, immune system, and pain response via the gut-immune-bone axis. This narrative review highlights a recent update on mechanistic evidence that bioactive is demonstrated demonstrated to reduce osteoarthritis pathophysiology.

## 1 Introduction

Osteoarthritis (OA) is an age-related degenerative disease severely impacting bone health. The OA burden is prevalent globally (1) and also rapidly increasing in India in the past decade (2). The OA mainly contributes to activity limitations and burdens the Nation’s effective workforce and healthcare (3). Moreover, it has recently been reported to affect the young (4) due to several underlying risks, including obesity, dietary, lifestyle, environmental transitions, nutritional imbalances, gut dysbiosis, metaflammation, and chronic diseases. The risk of developing osteoarthritis can be reduced and prevented by managing these factors. However, the global emergence of the metabolic syndrome (5) led to an incremental burden that forced us to comprehend systemic inflammation and immunity before the symptoms influenced and directed toward osteoarthritis pathophysiology. Musculoskeletal disorders (osteoarthritis, rheumatoid arthritis, osteoporosis) are targets of unresolved inflammation associated with the metabolism of long bone tissues. The interaction between bone remodeling and inflammation is continuous and modulated by immune response. A thorough understanding of the disease associated with osteogenic changes, immunomodulation, inflammation, and pain could be a basis required for holistic therapeutic interventions.

Despite OA being a multifactorial disease, weight management remains a primary focus for its prevention. The prevalence of OA brought community awareness and frequent check-ups to detect the disease at its early stages. Treatment of OA includes dietary modifications, lifestyle changes, analgesics, intra-articular preparations, and surgical procedures (6). The non-steroidal anti-inflammatory drugs (NSAIDs) and corticosteroids are used with potentially serious side effects. Thus, alternate therapeutics delay and/or reduce the progression of chronic inflammatory diseases like osteoarthritis is warranted. Nutrition, in maintaining healthy bone and musculoskeletal health, in general, has led to the emergence of Bioactives, nutraceuticals, botanicals, or herbal formulations, helping delay the onset of the disease and sometimes treating conditions. Functional foods and supplements may help the immune system as their secondary metabolites can target molecular pathways, ameliorating the disease. The pathogenesis of bone disease is associated with disrupting the osteoclasts-osteoblasts balance that governs bone remodeling. With their bioactive properties and ability to intervene in signaling pathways, nutraceuticals promise to help restore this balance (7–9). Molecular mechanisms that may define the underlying benefits of dietary bioactive against inflammatory diseases are limited. Bioactivity can inhibit TLR4 (Toll-like Receptor 4) mediated inflammation by modulating the gut microbiome (10). Dietary bioactive takes on the intestinal flora diversity, and their release of metabolites, activates immunomodulation by receptor signaling, transmits a peripheral signal, modulates nociception, pain, and systemic inflammation, and alters blood parameters, tissue metabolism, and cytokine profile, as demonstrated by several studies (discussed later).

Although the functions of bioactive compounds influencing osteoarthritis pathophysiology are reported, their modulation of the intestinal microbiome via the gut-bone axis is not known. In addition, there is paramount evidence that gut microbiota modulates host immune responses in osteoarthritis; however, the exact mechanisms are still unknown (11). In that context, this narrative review highlights recent mechanistic evidence on using bioactive or functional foods to ameliorate osteoarthritis and inflammatory diseases. In this article, Bioactives and their roles in bone metabolism of osteoarthritis are proposed based on available data and evidence. A comprehensive literature review was conducted using PubMed, Scopus, and Science Direct databases. The tenure of the search ranged from 2009 to 2023. The main aim of this review is to describe the use of Bioactives in OA, highlighting their molecular mechanisms of action.

### 1.1 Osteoarthritis risk, pathophysiology, intervention, and scope of research

Obesity, mainly upper body adiposity, poses a significant risk of developing musculoskeletal disorders like osteoarthritis (12). The epidemiological data suggested that women suffering from obesity and people with a sedentary lifestyle had a higher prevalence of osteoarthritis (13). Obesity, diabetes, and osteoarthritis are interlinked, with the latter being a predominantly acquired risk factor due to excess adiposity, increased mechanical load, loss of muscle mass, and low-grade inflammation modulated by adipokines. Type 2 diabetes (T2D), the most common form of diabetes, is a significant consequence of physical inactivity and obesity (14). Glucose intolerance and resistance to insulin developed during obesity result in various metabolic disorders, including osteoarthritis. The most detrimental effect of obesity is the mechanical load on weight-bearing joints, leading to the progression of osteoarthritis (15). Studies indicate that adiposity leads to low-grade inflammation, stress on weight-bearing joints, and upregulates leptin, which can regulate chondrocyte apoptosis. Distinct differences are visible between normal bone and cartilage, and these features are influenced by osteoarthritis (16). Normal cartilage exists in an avascular state, which maintains the integrity of the tissue. However, cartilage no longer remains in the avascular, and neovascularization occurs in a diseased state. Chondrocyte clusters and osteophyte formation also occur (17). The cartilage matrix also degenerates owing to increased hydration and loss of proteoglycans, decreasing keratan sulfate and hyaluronic acid. The resultant mechanical stress leads to surface fissures in the articular cartilage (4, 18).

Both systemic and localized inflammation are associated with the progression of osteoarthritis (19, 20), where several pro-inflammatory cytokines and matrix metalloproteinase (MMPs) are involved at both early and late stages of osteoarthritis. Interleukin (IL)-1β, IL-6, TNF-α, matrix metalloproteinase (MMP)-1, MMP-3, and MMP-13 are secreted mainly by chondrocytes (21). Diseased chondrocytes and synoviocytes also express IL-1, type-1 IL-1 receptor, and type II collagen. The OA progression correlates with inflamed synovium, typically characterized by infiltrated neutrophils to the affected site, mediated by several small chemotactic proteins or chemokines, such as IL-1β, IL-6, IL-8, and others. Elevated IL-6 (22) and IL-8 (23) levels were reported in synovial fluid from OA patients compared to controls. These chemokines are known to induce chondrocyte hypertrophy and show an increased risk of cartilage loss. OA progression is also mediated by cross-talk between growth factors and chemokine productions. Connective tissue growth factor (CTGF or CCN2) is also involved in several events of skeletogenesis by acting as a molecular bridge for extracellular matrix (ECM) by modulating angiogenesis, chondrogenesis, and osteogenesis (24). CTGF acted as a pro-inflammatory mediator by producing IL-6 (25), a pro-angiogenic modulator by producing IL-8 (26), and other chemokines. CTGF is abundantly expressed in the OA (27), but its pathogenic role is unclear. OA synovium in obesity is enriched with excess T-cells and B-cells, where specific cartilage turnover markers are also elevated in obesity compared with healthy (28). Thus, a detailed understanding of mediators and pathways that impact initiation to the progression of osteoarthritis pathophysiology is required to design targeted therapies for its intervention.

Bone remodeling and bone loss are controlled by a balance between TNF-family protein, osteoprotegerin ligand (OPGL), and its decoy receptor osteoprotegerin (OPG), preventing RANKL (receptor activator of nuclear factor kappa β ligand) from binding to and activating RANK (receptor activator of nuclear factor kappa β). The OPGL receptor, RANK, is expressed on osteoclast precursors, chondrocytes, and mature osteoclasts. The binding of RANKL to RANK leads to osteoclastogenesis and inhibits osteoclast apoptosis (29). Thus, balancing activities of myeloid origin bone-resorbing cells, i.e., osteoclast with mesenchymal origin bone-forming cells, osteoblast is modulated by RANKL and OPG actions. A dysregulated RANK-RANKL-OPG system increases RANKL activity associated with osteoporosis and secondary inflammation of bone disease (30). It has been demonstrated that the RANKL/OPG ratio is consistently elevated in inflammatory diseases. The OPGL regulates lymphocyte development, organogenesis, and interactions between the immune system’s dendritic cells and T-cells. Studies suggest activated T-cells directly trigger osteoclastogenesis through OPGL and RANK (31). Thus, modulators of T-cell activators could influence bone metabolism by regulating OPGL production and bone loss.

The available treatment for osteoarthritis includes corticosteroid injections like methylprednisolone acetate and triamcinolone, hyaluronic acid injections, topical and oral non-steroidal anti-inflammatory drugs, opioids, and surgical procedures like knee arthroscopy (32). While corticosteroid injections and surgical procedures carry a significant risk of infection and possible hyperglycemia, NSAIDs and opioids can result in ulcers, gastrointestinal perforation, and iatrogenic addiction (33). Recent studies have shown the possible usage of regenerative medicine in treating osteoarthritis (34). These include platelet-rich plasma injections, proliferation therapy, and stem cell therapy. Mesenchymal stem cells aid cartilage tissue regeneration by upregulating growth factors, reducing the inflammatory response, and differentiating into chondrocytes (35). However, significant concerns loom about biosafety and disease risk over stem cell-based therapy for osteoarthritis. These include the possibility of tumor formation, immune rejection, formation of ectopic tissue, or differentiation into undesired cell types (36). Bioactive enriched nutraceuticals and functional foods demonstrated early evidence to relieve osteoarthritis (37), and promised NSAID-like effects in ameliorating pain and function (38). The ability of bioactive to function similarly to that of NSAIDs bereft of potential side effects and thus can be exploited to delay disease progression. However, current research is still in the early stages, and it is essential to understand the underlying mechanisms in the bioactive-mediated amelioration of osteoarthritis.

## 2 Molecular pathways involved in osteoarthritis pathophysiology

In a chronic inflammatory state, particularly in rheumatoid arthritis, persistent inflammation results in bone loss by volume and mass (39) with a concomitant increase in local RANKL expression. Excess pro-inflammatory cytokines, including IL-6, IL-1β, and TNF-α produced by osteoclasts and osteoblasts, triggered the bone microenvironment by boosting RANK expression and bone resorption phenotype (40). The interplay of bioactive mediators and target cells could shape the inflammatory and metabolic control of bone remodeling. Therefore, activator and resolution of inflammation can impact bone metabolism and disease.

Understanding molecular pathways associated with osteoarthritis ensures an understanding the underlying reasons for disease pathology. Various pathways play a key role in the onset and progression of osteoarthritis, including Wnt/β-catenin, transforming growth factor β (TGF-β), bone morphogenic protein (BMP), fibroblast growth factor (FGF) signaling, nuclear factor κB (NF-κB) pathway, transient receptor potential vanilloid 1 (TRPV1) pathway, and RANK/RANKL/OPG pathway. In addition, various transcription factors and regulators involved in osteoarthritis progression include RUNX2 (Runt-related transcription factor 2), ADAMTS (a disintegrin and metalloproteinase with thrombospondin motifs), mTOR (mammalian target of rapamycin) and MMPs (41).

### 2.1 RANK/RANKL/OPG pathway

The role of RANK/RANKL/OPG and Wnt/β-catenin signaling in osteoarthritis has been extensively studied (42). This diseased state results in the alterations of both Wnt/β-catenin and RANK/RANKL/OPG signaling, disrupting the homeostasis of bone remodeling and upregulating pro-inflammatory cytokines. RANKL activates RANK (43), while M-CSF (macrophage colony-stimulating factor) and RANK initiate osteoclast differentiation from hematopoietic stem cells (HSCs). The interaction between RANKL and RANK also activates NF-κβ and MAP kinases. On the other hand, OPG, an antagonist, inhibits the binding between RANK and RANKL, keeping the system in check. TNF-α, IL-6, and IL-10 are essential cytokines in regulating bone remodeling. While osteoclasts differentiate from HSCs, osteoblasts differentiate from mesenchymal stem cells via osterix and Runx2 (runt-related transcription factor-2). Runx2 is regulated by bone morphogenetic proteins (BMPs) and Wnt proteins. Several growth factors and hormones, including PGE2, bFGF, parathyroid hormone, glucocorticoids, TGF-β, estrogen, and vitamin D3, also modulate bone remodeling.

### 2.2 Wnt/β-catenin pathway

The Wnt (wingless-related integration site) signaling pathway plays a crucial role in the growth and proliferation of skeletal tissues and bone development. β-catenin is a transcriptional co-activator that regulates the transcription of Runx2 and other transcriptional factors. An inactive Wnt pathway results in the proteasomal degradation of β-catenin, hence inhibiting transcription of the Wnt target genes (44, 45). Inactive Wnt proteins are usually bound to sFRP3 (secreted frizzled-related protein 3). In the active state of Wnt signaling, the destruction complex degrades β-catenin, is inactivated, and cellular β-catenin levels rise. β-catenin then translocates and binds to the TCF (T-cell factor), activating the transcription of Wnt target genes. Canonical Wnt signaling is directly involved in osteoblastogenesis from MSCs. The signaling pathway maintains balance during bone remodeling by promoting bone formation via osteoblasts. The dysregulation of several Wnt signaling factors and their extended crosstalk with other signaling pathways could lead to a diseased state.

### 2.3 NLRP-3 inflammasome and Nrf2/HO-1 signaling

The relationship between NLRP-3 (NOD-, LRR- and pyrin domain-containing protein 3) inflammasome and Nrf2 (nuclear factor erythroid 2-related factor 2) signaling has been demonstrated in the osteoarthritis (46). In this state, human synovial tissues revealed a higher expression of Nrf2, HO-1 (heme oxygenase 1), NLRP-3 inflammasome, and ASC, suggesting increased oxidative stress. Nrf2 knock-down in rat chondrocytes showed an increased expression of NLRP-3 inflammasome, suggesting its role in NLRP-3 activation caused by increased oxidative stress during the diseased state (46). Hence, both Nrf2 and NLRP-3 could be therapeutic targets for the treatment of osteoarthritis. Several studies suggest the involvement of NLRP3 inflammasome in various bone ailments, activating pro-inflammatory cytokines. Osteoarthritis’s pathogenesis involves abnormal activating proinflammatory cytokines (47) (48), in which IL-1β primarily plays an important role. While stimulating RANKL-osteoclast activation, IL-1β inhibits osteoblast differentiation, resulting in increased bone resorption and reduced bone formation. During this process, IL-1β modulates NF-κB and MAPK (mitogen-activated protein kinases) pathways. NLRP3 inflammasome is also involved in gasdermin D-associated cell lysis and death & pyroptosis. Reactive oxygen species also upregulate NLRP3-mediated pyroptosis.

### 2.4 TRPV1 pathway

Transient receptor potential vanilloid subtype 1 (TRPV1) is majorly expressed in nerve fibers that involve the regulatory role of cardiovascular, respiratory, and digestive systems and play an essential role in specific diseases (49). Although capsaicin is a known agonist of TRPV1, various other mediators such as prostaglandin, nerve growth factor (NGF), substance P, physical and chemical features such as acidic pH, osmotic pressure, redox state, and noxious heat stimulation, all lead to activation of TRPV1 (50). Targeting the TRPV1 channel pathway to alleviate osteoarthritis pain is effective based on the current data. Several antagonists and agonists of TRPV1 are currently under various phases of preclinical and clinical trials (51). In both primary mouse chondrocytes and surgically induced-DMM (destabilization of medial meniscus) mice OA model, single cell RNA sequencing revealed that TRPV1 led to upregulation of selenoprotein glutathione peroxidase 4 (GPX4) and showed protective effective against ferroptosis of chondrocytes (52). Receptors of TRPV1 were detected in the synovium of osteoarthritic joints, following which an antagonist of TRPV1 was administered locally. Blockage of TRPV1 led to significant inhibition of joint pain and proved to be a promising therapeutic against osteoarthritis (53). Various agonists of TRPV1, such as capsaicin, resiniferatoxin, allicin, gingerol, and piperine, have been shown to interact and bind to TRPV1 (54, 55). Several clinical studies have shown the effect of topical or intra-articularly administered capsaicin in alleviating OA-related pain (56, 57). An analog of capsaicin, pellitorine extracted from *Tetradium danielli* is an antagonist of TRPV1 and can thus inhibit pain (58). Intra-articular treatment with resiniferatoxin, another agonist of TRPV1 led to reduced pain in the induced osteoarthritis model (59).

Available data suggest modulators of the TRPV1 channel pathway could be the target in ameliorating OA-related pain. Thus, the Bioactives that interact with the receptor are potential therapeutic targets.

## 3 Mechanisms of action of Bioactives on osteoarthritis: gut-immune-bone axis

Poor nutrition or nutritional deficiencies have a severe adverse impact on the health of bones. Diet plays a pivotal role in the pathogenesis of osteoarthritis, where obesity is a high-risk factor. Dietary changes influence the gut microbiome, affect body weight, and cause obesity (60). However, the interplay between diet, body weight, obesity, and gut microbiota in influencing gastrointestinal, metabolic effects, and immune modulation contributing to OA pathophysiology is unclear. Various studies show a bidirectional aspect where gut dysbiosis leads to obesity and vice versa, where obesity results in gut dysbiosis (61–63). The gut consists of a highly diverse composition of microorganisms that govern and modulate a wide range of functions like digestion, metabolism, absorption of nutrients, vitamin synthesis, signaling pathways, inhibiting the growth of pathogenic bacteria, immunity and interaction with sensory afferent fibers releasing neurotransmitters, forming a gut-brain axis. The various roles the intestinal microbiome plays in modulating the immune system and their modulation by bioactive is not understood yet.

The gut microbiome is majorly involved in directly interacting with the immune system based on diverse stimuli, generating an immune response, activation of specific immune cells, production of metabolites, acting as a barrier via tight junction proteins, maintaining homeostasis of the microbial composition, and recognizing commensal microbes of the intestine. Hence, the seemingly simple gut microbiome has vast involvement in the immune system at both innate and adaptive levels (64–66). Dietary habits constitute a major causative for maintaining gut homeostasis. The bioactive that are proven to have various health benefits modulate the microflora composition and affect the immune system in case of inflammatory disorders. With respect to osteoarthritis, the innate immune system plays a crucial role in inducing inflammatory responses during disease progression (67).

The most commonly found microbial phyla include Firmicutes, Actinobacteria, Proteobacteria, Bacteroidetes, Fusobacteriia, and Verrucomicrobia, among which Bacteroidetes and Firmicutes represent almost 90% of the microflora (68). However, variations in the gut microbial composition are found in different parts of the gastrointestinal tract, in addition to variations in age and environmental factors. Among individuals, these variations arise from BMI, diet, exercise, ethnicity, climate, and geographical location changes. The ratio of Firmicutes to Bacteroides increases during obesity and is a characteristic of gut dysbiosis (69, 70). Increased abundance of Firmicutes during obesity leads to increased production of short-chain fatty acids (SCFAs) and increased concentration in the samples that contribute to reduced energy expenditure (71), resulting in expanded body weight (72). Dysbiosis in the gut changes the metabolic pathways of certain microbes, leading to how lipids and carbohydrates are metabolized (73). High-fat diet-induced obesity also has a risk of developing low-grade inflammation (74, 75) that causes gut dysbiosis and disrupts the homeostasis of intestinal permeability by altering the expression of tight-junction proteins (76) leading to a leaky gut with increased plasma levels (77) of lipopolysaccharide (LPS).

**Figure 1:**
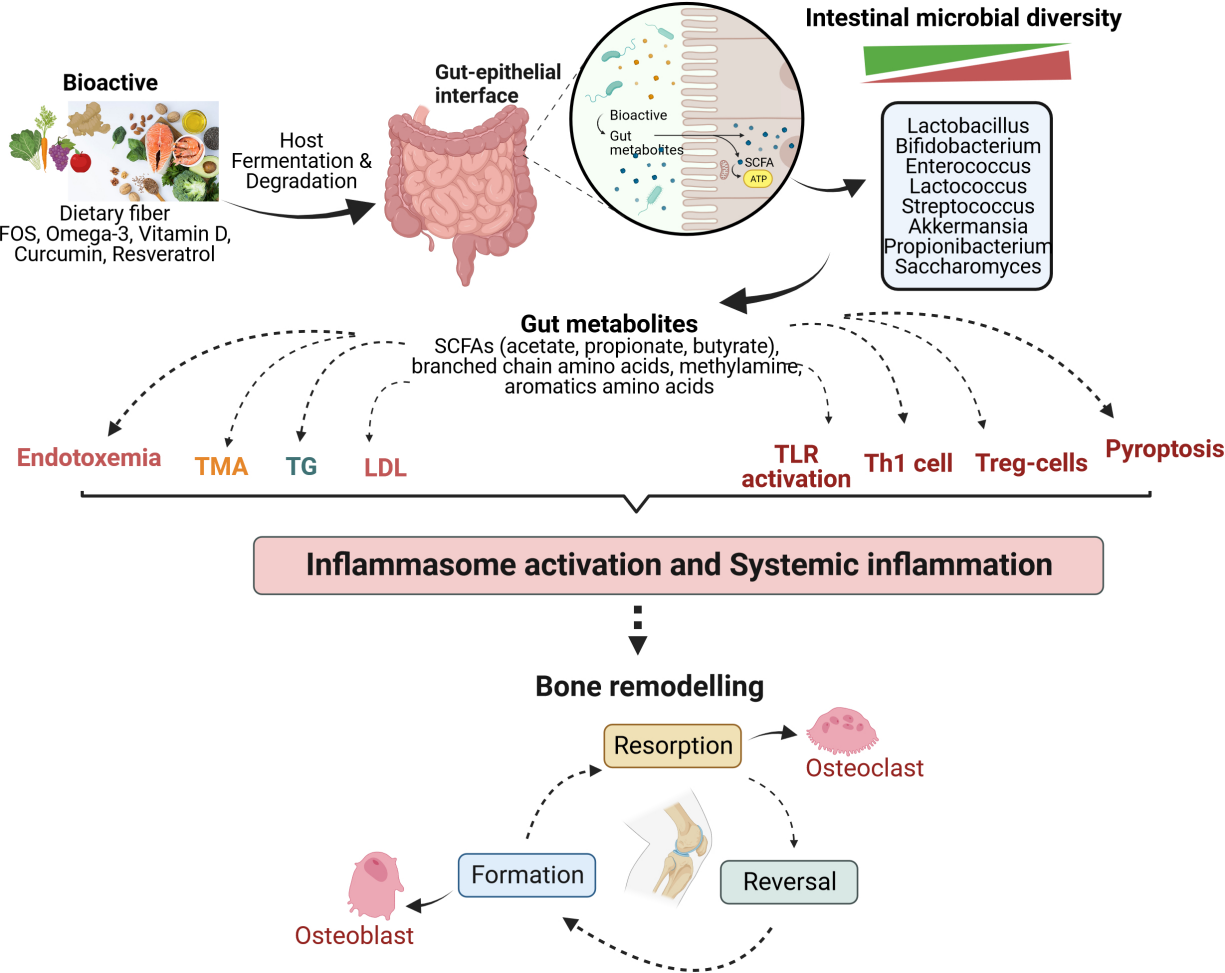
Mechanism of bioactives actions in modulating the bone metabolism of osteoarthritis by gut-immune-bone axis. Various bioactive compounds undergo fermentation in the host gut by intestinal microbiota, thereby regulating microbial diversity and generating metabolites that majorly modulate the immune system, among other significant functions. They are involved in lipid metabolism, influencing the levels of triglycerides and low-density lipoprotein. Gut metabolites also lead to differentiation and activation of immune cells to strengthen the intestinal barrier and maintain gut homeostasis. These mechanisms act at the system level, reducing inflammation and affecting bone remodelling to balance osteoclastogenesis (bone resorption) and osteoblastogenesis (bone formation). FOS, Fructooligosaccharide; SCFAs, Short chain fatty acids; TMA, Trimethylamine; TG, Triglyceride; LDL, Low density lipoprotein; TLR, Toll-like receptor; Th1, Helper T lymphocytes type 1; Treg, Regulatory T lymphocytes.

The immune system influences the gut composition and develops tolerance towards non-pathogenic microbes from the early stages. Gut microbiota affects the development and maturation of various immune cells. Together, the immune system and the gut microbiota also act as a barrier at the intestinal lining against pathogenic microorganisms (78) (79) (64). Various luminal factors like microbial metabolites (SCFAs, tryptophan metabolites, succinate, vitamins, and lipid mediators), dietary ω-3 fatty acids, sphingolipid, and specific neuropeptides play an active role in the innate immune system of the intestine (80). In addition, the prevalence of the gut-immune-bone axis has also been demonstrated by several studies (81–84). TLRs have been a significant link between the gut microbiome and immune responses generated by the immune system. Specific microbial metabolites of *C.sporogenes* regulate intestinal permeability by directly interacting with xenobiotic receptors via TLR4 signaling (85). However, it has been reported that a high-fat diet leads to an increase in pro-inflammatory cytokines, causes changes in the gut microbial composition, increases the abundance of *Enterobacteriaceae*, which enhances endotoxin production, increases intestinal permeability, and induces TLR4 expression (86). This is because TLR4 is a critical protein that regulates gut permeability and is involved in acute and chronic inflammation (87, 88). Certain *Prevotella* species in the intestine (*P. melaninogenica and P. copri*) are involved in inducing the production of a subset of T helper cells (Th17) via their interaction with TLR2 (89). In addition to TLR-4 and TLR-2, TLR-1 is also actively involved in maintaining intestinal homeostasis and obstructs the translocation of microbes, which otherwise happens during either defective TLR-1 or an inflammatory state (90). Thus, abnormal TLR signaling negatively impacts the gut, compromising permeability and resulting in inflammation and dysbiosis. A schematic outline (**Figure 1**) suggests an emerging mechanism that Bioactives could play their roles in bone metabolism of osteoarthritis by gut-bone axis.

## 4 Bioactives and osteoarthritis: metabolic and anti-inflammatory functions

The Bioactives that include probiotics and prebiotics have recently been examined extensively for their efficacies and potential health benefits due to minimum side effects when included in the diet of healthy adults. The primary focus of the ongoing research is the gut microflora’s relationship to chronic diseases. In general, probiotics are food such as yoghurt, curd or supplements that contain live microbial strains such as *Lactobacillus*, which can help to maintain healthy microflora in the body. In contrast, prebiotics are foods rich in Bioactives, including polysaccharides, fructooligosaccharide, polyphenols, omega-3 fatty acids and other that typically serves foods for endogenous gut microflora.

### 4.1 Dietary habit, gut microbial diversity and osteoarthritis

It has been reported that gut dysbiosis worsens osteoarthritis (91). A causal relationship has been established among microbial taxa such as *Methanobacteriaceae, Ruminiclostridium, Desulfovibionale*s and osteoarthritis (92). Knee osteoarthritis increases the abundance of microbes such as *Peptococcus*, *Propionibacterium*, *Intestimonas*, *Parvimonas*, and *Shimwella* (93). OA-related knee pain has been shown to correlate with the abundance of *Streptococcus* and *Clostridium* positively and reduced α- and β diversity (94) (95). A decrease in species of *Bifidobacteria* and *Faecalibacteria* is also observed. However, precise mechanisms that would increase the potential of gut microbiome being targeted to maintain immune homeostasis and prevent, delay, or treat inflammatory disorders like OA are required to investigate further.

The gut microbiome varies among populations from diverse geography, considering their subsistence and dietary patterns, ethnicity, and genetic factors (96). The urban Chinese population has comparatively less α and β-diversity and a decreased ratio of archaea-to-bacteria compared to the rural population (97). *Methanobrevibacter smithii* was abundant in rural populations, correlating with other SCFA-producing bacteria like *Prevotella* and *Roseburia* sp. Geography played a bigger role than ethnicity in influencing gut microbial composition in China (98). In India, across several regions and varied ethnic groups, the composition was dominated by Bacteroidetes, with *Prevotella* involved in carbohydrate metabolism being the most abundant (99, 100). Dietary patterns were also seen to bring in variations in the composition among different regions. Upon comparative analysis of the gut microbial composition of Indians with people from the United States, France, Germany, and Denmark, it was revealed that *Prevotella*, *Bifidobacterium*, and *Lactobacillus* were more abundant in Indians than people from these countries (100). Among the Spanish, Firmicutes and Bacteroidetes were the most dominant phyla, in which *Faecalibacterium, Bacteroides, Alistipes, Oscillospiraceae*, and *Prevotella* were prevalent at the genus level (101). Age, BMI, and diet-dependent associations with several human gut microbes were also noted. In Italy, variation in gut microbial composition was evident among samples from different geographical regions. Cyanobacteria and Nitrospirae were found in all samples of two individually separate regions (102). Still, they were absent in the other areas, proving the association between gut microbial composition and geographical location. Humanized gnotobiotic mice from various geographical locations (US, Fiji Islands, and Guatemala) had variations in their susceptibility to enteric infection caused by *Citrobacterium rodentum* (103). This establishes that geographic differences have a role in influencing health status by modulating gut variations. So far, studies have shown that Firmicutes and Bacteroidetes form the most dominant phyla, irrespective of geographical differences. However, a latitude-based correlation has been noted in affecting the Firmicutes-to-Bacteroidetes ratio. Europeans were abundant in Firmicutes, which decreased in the American, African, and Asian populations. On the contrary, Bacteroidetes were dominant in African and American and drastically reduced in European (104). Upon comparative analysis of the gut microbial composition among the obese population of France, French Polynesia, Amazonian French Guiana, and Saudi Arabia, it was revealed that the gut microbiome of obese Amazonians varied drastically compared to other populations because of different cultural and dietary habits (105). Consuming food rich in fiber, starch, and polysaccharides from plants led to increased microbial diversity in Polynesians and Amazonians compared to French and Saudi obese populations. Spirochaete*s* and specific *Lactobacillus sp* abundant in Amazonians were absent in other obese populations.

### 4.2 *Polysaccharides, fructooligosaccharide* and osteoarthritis

Since polysaccharides resist digestion, they are subjected to gut microbial fermentation. The beneficial effects of bioactive polysaccharides depend on their fermentability by gut microbiota, water-holding capacity, bile acid interaction, and other physicochemical properties. These bioactive slows down gastric emptying, acidosis, improving bowel function by modulating the gut microbiota structurally and functionally and thus protecting the immune system. Bioactive polysaccharides reshape intestinal microbial activity through bacterial fermentation. A healthy human gut microbiome abundantly expresses carbohydrate-active enzymes (CAZymes) essential in metabolizing specific complex polysaccharides like dietary fiber (106). The dietary shifts in infant from milk to solid food usually change the CAZymes (107). Varied dietary habits across geography and lifestyle contribute to variations in CAZymes. An Italian cohort compared the microflora of healthy and obese type 2 diabetes patients and found that healthy subjects who consumed a Mediterranean diet had more abundance of CAZymes-producing bacteria like *F. prausntiznii and E. rectale*. In contrast, the obese subjects suffering from T2D showed an abundance of *R. bromii and S. variabile* that would result in dysregulation of the gut (108). Adding dietary fiber or complex polysaccharides into the diet promotes the abundance of bacteria that can ferment it and use it as an energy source while generating various metabolites that prove beneficial for the hosts in terms of reducing inflammation via gut modulation. However, a refined diet can make these microbes redundant and alter the microbiome (109). Supplementation of dietary fiber in healthy adults increased the abundance of Bacteroidetes, thereby changing the ratio of Bacteroidetes: Firmicutes (110). A multicenter cohort suggests that intake of the recommended daily dietary fiber was associated with a reduced risk of knee pain over time (111). The prospective US cohorts over four and nine years showed that dietary fiber reduced the risk of developing symptomatic but not radiographic osteoarthritis (112). However, much work is required to emphasize how dietary fiber can reduce the risk of symptomatic osteoarthritis (113).

Supplementation with fructooligosaccharide (FOS) purportedly improves the concentration of *Bifidobacteria* and increases the function of dendritic cells (114). FOS supplementation in colitis-induced mice led to the manipulation of intestinal flora, reduced the abundance of *Mucispirillum*, downregulated expression of pro-inflammatory cytokines (TNF-α and IL-6) and upregulated IL-10 expression (115). FOS intake improved immune responses by ameliorating the hamster’s HFD-induced inflammation and lipid profile (116). FOS supplementation affected Treg and Th17 cells’ homeostasis using tryptophan metabolites and modulating the gut microbial composition (117). The *in vitro* fermentation of chicory (FOS) and native inulin utilizing pooled fecal inocula of infants exhibited reduced pro-inflammatory cytokines produced by immature dendritic cells via increased synthesis of succinate and lactate (118). A combined prebiotic and probiotic (*Bacillus coagulans*) supplementation in mice treated with cyclophosphamide showed increased expression of anti-inflammatory cytokines such as IFN-γ and IL-4 where the abundance of several beneficial microbes such as *Enterococcus, Bacteroides, Ruminococcus, Oscillospira*, and *Anaerotruncus* was found to increase (119). Prebiotic fiber like FOS showed an increased abundance of *B. pseudolongum*, a *Bifidobacterium* that is ablated during obesity, while reducing the number of microbes from *Peptococcaceae* family and other species associated with obesity, thereby reestablishing the microbiome associated with a lean gut (120). In addition, supplementation with oligofructose in obese mice with surgically-induced osteoarthritis led to reduced synovial inflammation, reduced mineralization of the meniscus, and downregulated chondrocyte hypertrophy (120).

Prebiotic supplementation in the form of short-chain galactooligosaccharides, long-chain FOS, and milk oligosaccharide 2’-fucosyllactose at early life in mice improved their immune response towards vaccine by generating specific antibodies, promoted the production of cytokines and variation in the relative abundance of various microbial phyla. Prebiotics led to an increase in the quantity of Actinobacteria and a decrease in Proteobacteria and Firmicutes (121). A specific prebiotic blend (mixture of anthocyanins, inulin, FOS, and galactooligosaccharides) was found to reduce pro-inflammatory cytokines and upregulated the expression of tight junction protein *in vitro* and *in vivo* models of inflammatory bowel disease (122). Prebiotic blend increased the relative abundance of specific genera such as *Prevotella, Intestimonas, Butyricicoccus, Barnesiella*, and *Ochrobactrum*. Supplementation of prebiotics (galactooligosaccharides and fructooligosaccharides) and postbiotics in healthy suckling rats resulted in specific changes in gut microflora composition and immune responses. Prebiotics led to variation in the composition of an increased abundance of *Peptostreptococcaceae, Anaerotruncus*, and *Romboutsia*, reduction in *Akkermansia* and *Vibrionimona*, altered the proportion of SCFAs and modulated the immunoglobulin profile (123). However, postbiotic supplementation alone and in combination with prebiotics increased the expression of Toll-like receptors such as TLR3, TLR4, TLR5, TLR7, and TLR9, indicating their role in generating an immune response.

The comprehensive systematic review suggests the benefit of antioxidant supplementation to reduce disease-related symptoms in knee osteoarthritis patients (124). Quercetin, an antioxidant used in the treatment of osteoarthritis, influenced rat metabolome and gut microbial composition by increasing *Lactobacillus* species and levels of SCFAs (125). The presence of *Peptostreptococcaceae* and *Desulfovibrio*, both of which hurt the intestine, and the absence of lactic acid-producing bacteria have been shown in rat models of osteoarthritis (126). Orally supplemented butyrate and *Lactobacillus acidophillus* (LA-1) ameliorated osteoarthritis in rats by downregulating inflammatory cytokines and improved autophagy by reducing necroptosis factors (126). In another study, LA-1 alleviated cartilage degradation and pain in the monoiodoacetate-induced osteoarthritis (127). A similar probiotic supplementation downregulated enzymes that degrade cartilage, inflammatory cytokines, and reduced expression of markers associated with pain in mice (128). The probiotic supplemented with *Lactobacillus casei* strain Shirota (LcS) showed improved WOMAC and VAS scores and significantly lowered C-reactive protein levels in knee osteoarthritis patients (129).

### 4.3 Polyphenols and osteoarthritis

Curcumin, one of the most researched nutraceuticals, has antioxidant and anti-inflammatory properties that modulate several pathways, including NF-κB, Wnt signaling, Nrf2 signaling, NOTCH, mTOR, and JAK/STAT (130). The role in alleviating osteoarthritis and protecting bone health due to its anti-inflammatory properties has been highlighted recently. The chondroprotective effects of curcumin alleviated apoptosis in chondrocytes upon IL-1β stimulation (131). Stimulating with IL-1β also caused increased p65 promoter activity of NF-κB, which curcumin reversed. In THP-1 cells treated with LPS and ATP, curcumin inhibited the expression of TNF-α and IL-1β in addition to downregulation of cleaved-caspase-1, thereby inhibiting NLRP3 activation (132). The increased stability and bioavailability of curcumin nanoformulation showed a protective effect on the articular cartilage in the OA model, which was visualized in the histological examination (133). The nanoformulation enhanced matrix staining and increased cellularity compared to native curcumin. Curcumin and nanocurcumin downregulate pro-inflammatory cytokines IL-1β, TNF-α. In the model, topically administered nanocurcumin slowed the progression of osteoarthritis, downregulated pro-inflammatory mediators, and showed chondroprotective effects and improved locomotor function (133). Oral curcumin was also effective in slowing the progression of osteoarthritis; however, it could not significantly reduce OA-related pain as compared to the nanoformulation (133). A clinical trial with curcumin nanomicelles significantly ameliorated osteoarthritis symptoms in knee patients over six weeks, which was analyzed through a questionnaire (134). Curcumin primed onto adipose-derived mesenchymal stem cells-derived small extracellular vesicle exhibited improved efficacy and chondro-protective effects against osteoarthritis by inhibiting oxidative stress and subsequent apoptosis of chondrocytes (135). In the anterior cruciate ligament transection surgery (ACLT) induced osteoarthritis mice, histological examination and oxidative biomarker assays confirmed reduced apoptosis of chondrocytes, thus showing protective effects on the cartilage when compared to only small extracellular vesicles and only curcumin *in vivo*.

**Figure 2:**
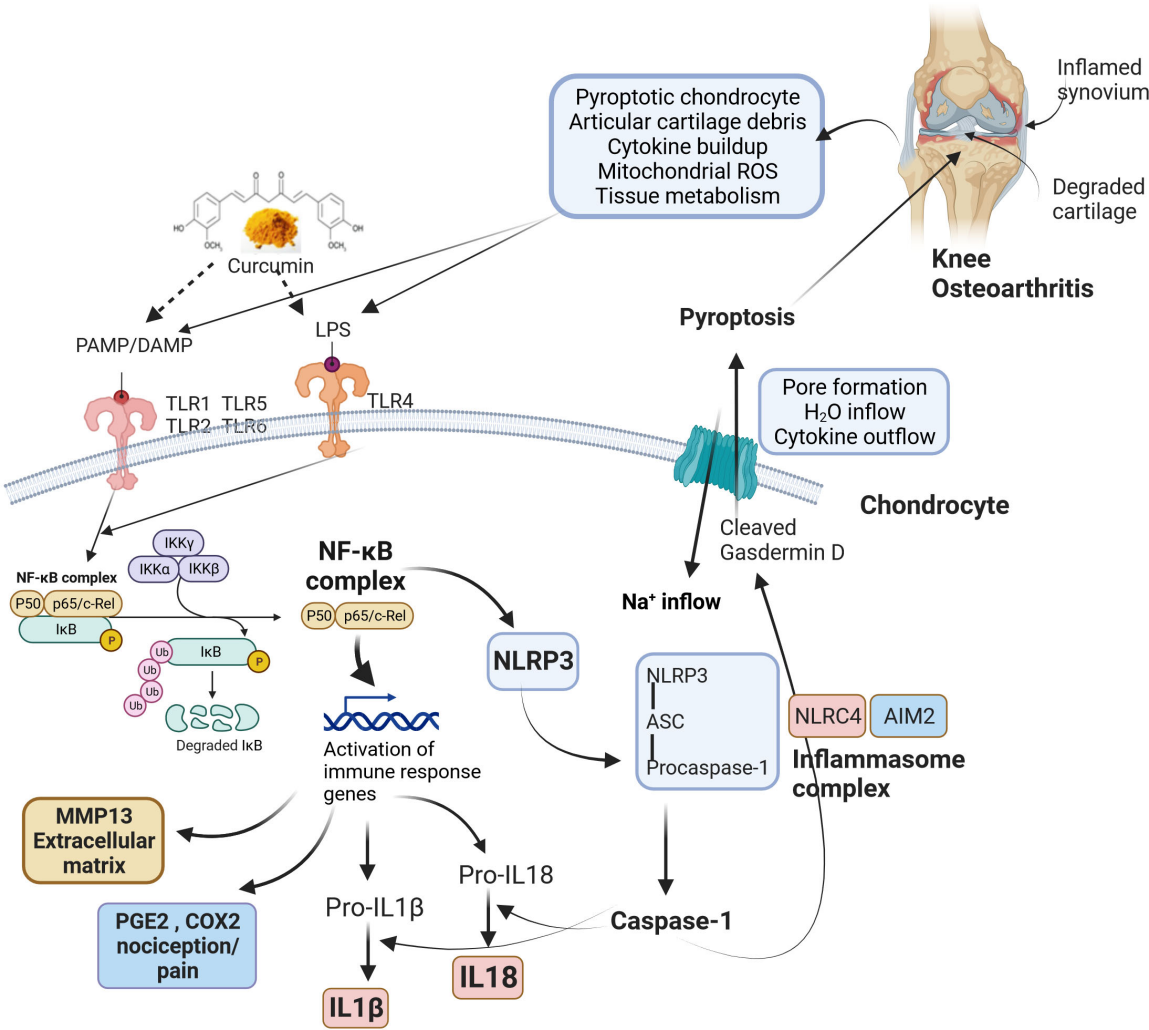
The roles of bioactive curcumin and modulation of osteoarthritis pathophysiology. Curcumin acts as a signalling molecule via PAMPs (pathogen-associated molecular patterns) and DAMPs (damage-associated molecular patterns) recognition that follows the activation of NLRP3 inflammasome, reduction of NLRP3-mediated inflammation, oxidative stress, possibly restoring the balance between osteoclastogenesis and osteoblastogenesis. Pathophysiological changes during knee osteoarthritis involve damaged cartilage tissue and inflamed synovium, which trigger various inflammatory pathways via PAMP/DAMP. The activated TLRs induce NF-κB signalling, leading to the upregulation of various genes related to immune responses. NF-κB complex also activates cytokines (IL-18 and IL-1β) and NLRP3. Activation of NLRP3 results in the formation of an inflammasome complex and cleaves pro-caspase 1 to caspase 1. Similarly, NLRC4 and AIM2 inflammasomes also form inflammasome complexes and activate caspase 1. Caspase 1 recognizes and cleaves Gasdermin D, producing transmembrane pore-forming channels that release pro-inflammatory cytokines. In addition, pore formation results in sodium and water inflow into cells. These factors cause programmed cell death (pyroptosis), further amplifying inflammation and aggravating knee osteoarthritis. ROS, Reactive oxygen species; LPS, Lipopolysaccharide; PAMP, Pathogen-associated molecular pattern; DAMP, Damage-associated molecular pattern; TLR, Toll-Like Receptor; NF-κB, Nuclear factor kappa B; IκB, I-kappa-B; IKK, Inhibitor of IκB kinase; MMP13, Matrix metalloproteinase 13; PGE2, Prostaglandin E2; COX-2, Cyclooxygenase 2; IL-1β, Interleukin-1β; IL-18, Interleukin 18; H2O, Water; Na+: Sodium; NLRP3, Nucleotide-binding domain, leucine-rich-containing (NLR) family, pyrin domain containing 3; ASC, Apoptosis-associated speck-like protein containing a caspase-recruitment domain; NLRC4, NLR family caspase activation and recruitment domain-containing protein 4; AIM2, Absent in melanoma 2.

The mechanism of bioactive curcumin in modulating osteoarthritis pathophysiology is elusive. Proinflammatory cytokines are involved in bone and joint-related diseases by inducing bone resorption and destroying cartilage (136). These cytokines are upregulated upon activation of NLRP3 inflammasome via PAMPs (pathogen-associated molecular patterns), DAMPs (damage-associated molecular patterns), and other signal molecules. Initially, NF-κB signaling is activated by toll-like receptors (TLR) upon recognition of PAMPs/DAMPs. This is called inflammasome priming and upregulates the expression of NLRP-3, pro-IL-1β, and pro-IL-18 (137). Post this, NLRP-3 forms NLRP-3 inflammasome, pro-caspase 1, and ASC. Pro-caspase 1 subsequently changes into caspase-1, which cleaves pro-IL-1β and pro-IL-18 into their active forms, such as IL-1β and IL-18. NLRP-3 inflammasome and NLRP-1 inflammasome induced pyroptosis in human primary fibroblast-like synoviocytes (FLS) collected from knee osteoarthritis patients under the stimulation of LPS/ATP (138). FLS cells, when stimulated with LPS and ATP, resulted in pyroptosis, which was, however, reversed in the presence of NLRP-1 and NLRP-3 siRNAs. The curcumin-ameliorating effects in patients with knee osteoarthritis symptoms could mediated by modulating NLRP3 inflammasome and its constituents that can effectively maintain the balance between osteoclastogenesis and osteoblastogenesis, reducing NLRP3-mediated inflammation oxidative stress and possibly improving osteoarthritis pathophysiology as shown in **Figure 2**.

Curcumin supplementation in mice showed neuroprotective effects by modulating the gut microbiota via the gut-brain axis (139). Curcumin was found to restore the profile of short-chain fatty acids and alleviated the expression of tight junction proteins of the intestine in comparison to diseased samples. Curcumin modulates the organization of tight junctions and downregulates LPS-mediated IL-1β expression, thus improving the intestinal barrier function (140). Supplementation with curcumin, Emblica, and Cassia extract in HFD fed-obese mice showed decreased intestinal inflammation and an improvement in the integrity of the gut epithelial barrier (141). Further, curcumin simultaneously reduced the plasma and mRNA levels of IL-1β and TNF-α downregulated the expression of MyD88, NF-κB, and TLR4 while upregulating major tight junction proteins such occludin and zonula occluden-1 in HFD-induced steatosis mice (142). Thus, there is strong evidence that dietary intervention with curcumin can improve the gut barrier function and potentially ameliorate inflammatory diseases.

Linking gut microbial composition, DHA-acylated curcumin diesters affected the abundance of microbes that metabolize trimethylamine and lipopolysaccharide. Thus, curcumin esters combined with DHA have significantly reduced renal tubal injury (143). Curcumin’s presence attenuated LPS-induced intestinal imbalance by reducing pro-inflammatory cytokine levels and impacting gut microbial diversity, leading to an increased abundance of *Enterococcus* and *Butyricicoccus* (144). Despite low absorption and low bioavailability, curcumin improves the function of the gut epithelial barrier via various mechanisms, including absorption into intestinal epithelial cells, binding to intercellular vitamin D receptor, direct interaction with IL-2, downregulation of sterol transporter NPC1L1, thus reducing intestinal cholesterol absorption (145). When combined with piperine, curcumin has also been shown to act on the mTORC inhibitor (TSC2), thereby downregulating mTORC signaling in human intestinal cells (146). Additionally, it has been shown that gut bacteria like *Blautia* sp. are involved in metabolizing curcuminoids into active metabolites such as demythylcurcumin, bisdemethylcurcumin, and demethyldemethoxycurcumin, all of which are promising synthetic analogs of curcumin that potentially show systemic and localized effects (147). Curcumin, thus, has the potential to modulate the epithelial barrier of the intestine (145). **Table 1** represents data from various clinical trials conducted to test the efficacy of curcumin against osteoarthritis. In addition, a meta-analysis has revealed that both high- and low-dose curcuminoids significantly decrease pain relief (172) and promise an alternative or complementary agent for the management of osteoarthritis (173).

**Table 1:** Clinical interventions with various bioactive supplemented in knee osteoarthritis patient.

Bioactives supplemented	Sample size, duration & measure	Key outcome	References
*Lactobacillus casei* Shirota	N=537, 6 mo -VAS score -WOMAC score -Serum parameters	Probiotic supplementation significantly improved VAS and WOMAC scores, reduced serum hs-CRP levels	(129)
Oral Vit D3 (50,000 IU/once/mo)	N=413, 2 yr -WOMAC score	No significant change in volume of tibial cartilage and WOMAC scores	(148)
Fish oil High dose (4.5g omega-3 @ 15ml/d Low dose (0.45g omega-3) @ 15ml/d	N=202, 2 yr -WOMAC score	Significant improvement in the low-dose group in WOMAC scores compared to high-dose	(8)
Resveratrol (500mg/d) plus, Meloxicam (15mg/d)	N=110, 3 mo -KOOS score -WOMAC score -Serum pro-inflammatory markers	Nonsignificant and weak correlation between serum pro-inflammatory markers and clinical scores i.e., KOOS and WOMAC	(149) (150)
Verbascox^®^ (Herbal extract) 800mg/d	N=100, 2 wk -Serum substance P markers	Improved functional capacity, reduced pain, and serum substance P levels, and equally safe as celecoxib (NSAID)	(151)
*Nigella sativa* oil (2.5ml/thrice/d)	N=116, 1 mo -WOMAC score -VAS score	Significant reduction in VAS and WOMAC scores	(152)
Ginger powder (500mg/d)	N=120, 3 mo -Serum markers	Reduced levels of IL-1β and TNF-α in serum	(153)
ParActin^®^ *Andrographis paniculata* wall (300mg/d and 600mg/d)	N=103,12 wk -WOMAC score -SF-36 -FACIT score	Significant reduction pain, stiffness and fatigue assessed by WOMAC, SF-36 and FACIT scores.	(154)
Burdock root tea (2g/150ml/thrice/d)	N=36, 6 wk -Serum markers	- Significant decrease in serum IL-6, hs-CRP - improved oxidative stress and inflammatory status	(155)
Blueberry Freeze-dried powder (40g/d)	N=63, 4 mo -WOMAC score -GAITRite^®^ -Serum markers	- Reduced stiffness and pain, improved gait performance and quality of life -Downregulated the expression of MCP-1	(156)
*Camellia sinensis* (green tea)	N=50, 4 wk -WOMAC Score -VAS score	Improved WOMAC and VAS pain scores	(157)
Marine bioactive (LD-1227)	N=60, 18 wk -VAS score -WOMAC score -KOOS score -Laquesne Index	Improved VAS, WOMAC, KOOS and Lequesne Index scores compared to control	(158)
Arthem^®^ (*Artemisia annua* extract) (150mg/twice/d)	N=34, 6 mo -WOMAC score	Significant improvement in WOAMC scores	(159)
Arthem^®^ Low dose -150mg/twice/d High dose - 300mg/d	N=42, 12 wk -VAS score -WOMAC score	WOMAC and VAS scores improved significantly in low dose group	(160)
Chicory root extract (600, 1200 and 1800 mg/d)	N=40, 4 wk -WOMAC	Improvement in stiffness and pain analyzed by WOMAC	(161)
Pomegranate Juice	N=38, 6 wk -WOMAC score -Serum markers	Reduced stiffness, decreased serum matrix metalloproteinases and improved antioxidant status	(162)
*Boswellia serrata* extract (Boswellin^®^)	N=48, 4 mo -Serum markers	Reduction in bone spurs, improved knee gap, reduced CRP level	(163)
Fennel seed extract (200mg/four times/d)	N=66, 2 wk -WOMAC score -VAS score	Reduced pain & stiffness, WOMAC and VAS score	(164)
Bromelain (500mg/d/16 wk)	N=40, 16 wk -WOMAC score -SF-36	Reduced SF-36 and WOMAC scores, however not significant when compared to diclofenac (NSAID)	(165)
Each capsule contains hemp seed oil (413mg), β-caryophyllene (35 mg), myrcene (15 mg), ginger extract in gingerols (66 mg) @ 2 capsules/d	N=38, 45 d -ODI score -SF-12 score	Various measures like Oswestry Disability Index (ODI) and Short Form 12 (SF-12) showed improvement in joint function and pain	(166)
Pycnogenol^®^ (Pine bark extract) 100mg/twice/d	N=33, 3 wk -Serum markers -Cartilage and synovial fluid	- Downregulated expression of cartilage degradation gene markers and IL-1β, MMP-13 and MMP-3. - Reduced serum concentration of ADAMTS-5	(167)
Garlic (1000mg/capsule/d)	N=76, 12 wk -WOMAC score	-Stiffness reduced significantly compared to placebo -WOMAC scores were insignificant	(168)
Garlic (1000mg/twice/d)	N=80, 12 wk -VAS score -Serum markers	-VAS scores reduced significantly - Serum resistin and pain scores decreased	(169)
Strawberry beverage (50g/d)	N=17, 26 wk - ICOAP score	-Reduced intermittent and constant osteoarthritis pain (ICOAP)	(170)
Strawberry supplement (50g/d)	N=17, 26 wk -Serum markers	-Decreased serum TNF-α in addition to reduced pain	(171)

mo, Month; wk, Week; d, Day; (VAS), Visual Analog Scale; (WOMAC), Western Ontario and McMaster Universities Arthritis Index; (AUSCAN), Australian/Canadian Osteoarthritis Hand Index; (OMERACT), Outcome Measures in Rheumatology; (OARSI), Osteoarthritis Research Society International; (KOOS), Knee Injury and Osteoarthritis Outcome Score; (ODI), Oswestry Disability Index and Short Form 12 (SF-12); Short Form 36 (SF-36); (FACIT), Functional Assessment of Chronic Illness Therapy; (ICOAP), Intermittent and Constant Osteoarthritis Pain; (IL), Interleukin; (TNF-α), Tumor Necrosis Factor-α; (CRP), C-Reactive Protein; (MCP), Monocyte Chemoattractant Protein; (NSAID), Non-Steroidal Anti-inflammatory Drug; (MMP), Matrix metalloproteinases; (ADAMTS), A Disintegrin and Metalloproteinase with Thrombospondin motifs.

Inflammation due to dietary habits like HFD consumption is a significant causative for intestinal dysbiosis. The bioactive polyphenols have the potential to rescue gut dysbiosis and maintain intestinal homeostasis. Resveratrol has demonstrated protective effects on systemic and localized inflammation by modulating the gut in several HFD-induced metabolic studies. Intervention with resveratrol in healthy rats led to changes in the gut microbiota composition like enrichment of *Parabacteroides, Bacteroides, Blautia, Lachnoclostridium, Ruminiclostridium*, and *Lachnospiraceae* (174). Further, transplanting these microbiomes to mice fed with HFD increased insulin sensitivity, improved gut barrier function, reduced weight gain, decreased inflammation, and improved lipid metabolism (174). Resveratrol reduced systemic inflammation and fat accumulation in mice fed with HFD. A correlation was observed upon fecal microbiota transplantation in mice from HFD-fed mice who intervened with resveratrol, thus proving that resveratrol affects the gut microflora composition (175). An increased abundance of *Ruminococcaceae, Akkermansia*, and *Lachnospiraceae* and reduced *Desulfovibriwere* mitigated HFD-induced hepatic fat accumulation, inflammation, weight gain, and gut dysbiosis (176). A combination of resveratrol and quercetin in HFD-fed rats led to reduced ratio of Firmicutes to Bacteroidetes, enriched with *Christensenellaceae, Ruminococcaceae, Bacteriodales, Akkermansia* and a decreased abundance with *Bilophila, Lachnoclostridium, Coriobacteriaceae, Acidaminococcaceae* and *Desulfovibrionaceae* (177).

Similar to curcumin, metabolites of resveratrol such as lunularin and dihydroresveratrol are derived from the gut microbiota and distributed abundantly in the GI tract and tissues as compared to unmetabolized resveratrol and hence show better activity than native resveratrol (178). Resveratrol exhibited cytoprotective, antioxidant, and anti-inflammatory properties when exposed to the gut microbiota-derived uremic toxin by downregulating Nrf2 and upregulating NF-κB expression in RAW 264.7 cells (179). Trans-resveratrol has been shown to upregulate mRNA expression of an occluding gene that expresses a tight junction protein and other inflammatory markers like TLR2, TLR4, and IL-18 (180). An increased abundance of SCFA-producing bacteria - *Dorea* and *Blautia* sp. and a reduction in disease-causing *Desulfovibrionaceae* sp. has been observed upon resveratrol supplementation in rats fed with an HFD (181). Thus, resveratrol’s anti-oxidative and anti-inflammatory potential is modulated via gut microbiome, as evidenced by several studies.

Various evidences suggest the established use of another polyphenol, capsaicin (CAP), as a phytochemical analgesic that works by binding with TRPV1, a nociceptive fibre (182–185). The vanillyl moiety of capsaicin is majorly responsible for its bioactivity and is used widely for the treatment of chronic musculoskeletal and neuropathic pain, chronic migraines and in treating functional dyspepsia (186, 187). Capsaicin also shows an anti-inflammatory effect by down-regulating LPS-induced expression of COX-2 and NF-κB in RAW 264.7 macrophages (188). Capsaicin has similarly been found to suppress the LPS-induced expression of pro-inflammatory cytokines TNF-α, IL-1β, and IL-6 in THP-1 macrophages (189). Capsaicin significantly reduced TLR4 and ICAM-1 expression in primary Schwann cells (190). In muscle precursor myoblast cells, capsaicin could attenuate LPS-induced inflammation by downregulating the expression of TNF-α, Calpain-1, and Caspase-3 (191). A nanoemulsion of capsaicin with olive oil proved to be a more efficient anti-inflammatory agent and analgesic upon topical application in rats and rabbits (192).

Dietary capsaicin has also been shown to alleviate metabolic dysregulation in diabetic obese mice, thus exhibiting anti-obesity properties (193). Several mechanisms in this regard have been explored (194). Capsaicin has satiety-inducing properties and suppresses appetite (195). It also decreases the accumulation of lipids by enhancing their metabolism (196, 197). Capsaicin also exhibits properties of improving glucose homeostasis and insulin sensitivity. Administration of dietary capsaicin in db/db mice (diabetic) led to increased glycogen synthesis and reduced gluconeogenesis in addition to increasing the pool size of bile acid by suppressing FGF15 and upregulating expression of glucagon-like peptide (GLP-1) (198, 199).

In addition, gut microbial changes have also been associated with the anti-obesity effects of capsaicin. A disruption in glucose homeostasis is linked to alterations in the gut microbiome. Dietary capsaicin increased the ratio of Firmicutes/Bacteroides and an increased abundance of *Roseburia* in obese diabetic ob/ob mice (200). Capsaicin led to an increased abundance of *Odoribacter, Coprococcus, Prevotella, Allobaculum*, *Muribaculaceae*, *Bacteroides*, and *Akkermansia*, while reducing the abundance of *Escherichia, Sutterella*, *Helicobacter, Proteobacteria* and *Desulfovibrio* (201, 202). This increase in SCFA-producing species in HFD mice had an anti-obesity effect by increasing propionate and acetate concentrations. A combination of dietary fiber and capsaicin in HFD-rats increased gut-microbial diversity, reduced the abundance of *Desulfovibrio*, and increased *Akkermansia* and *Allobaculum* species (197). This evidence led us to believe that capsaicin might also function in a TRPV1-independent way by modulating the gut.

The ability of capsaicin to further increase gut microbial diversity has also been elucidated. Microbial community was maintained *in vitro* and supplemented with capsaicin for two weeks. Analysis of samples revealed a distinguished increase in microbial diversity and a modulation in the abundance of primary short-chain fatty acids (SCFAs) (203). Dietary intervention with capsaicin led to lowered plasma Ghrelin levels and increased levels of gastric inhibitory peptide (GIP) and GLP-1. It also led to an increased abundance of *Faecalibacterium* while increasing the ratio of Firmicutes-to-Bacteroide (204). Additionally, the administration of capsaicin also led to a dose-dependent increase in the production of SCFAs by regulating the abundance of *Lactobacillus*, *Butryricimonas*, *Bifidobacterium*, and *Faecalibacterium* (205). *Faecalibacterium*, *Lactobacillus*, and *Roseburia* are essential species of the gut microbial community, all of which have been shown to increase in abundance upon administration of dietary capsaicin, proving their role in positively modulating the gut microbiota (206).

Randomized controlled trials (RCT) studied the efficacy of dietary supplementation in improving bone and skeletal health. A recent systematic review of ten studies confirmed that turmeric or curcumin extract improved pain and function from baseline for individuals with knee osteoarthritis (38). Other Bioactives also demonstrated beneficial effects in improving bone disorders. For example, a combination of protein fortified with micronutrients was given as a nutraceutical, and markers related to bone turnover and bone metabolism were studied in premenopausal women. A decline in the expression of bone resorptive markers and increased vitamin B levels showed a promising outcome for the formulated supplement against skeletal health (207). The healthy post-menopausal women supplemented with pomegranate and grape seed juice showed downregulated expression of genes related to bone resorption and osteoclast differentiation and upregulated bone formation-related genes in ex-vivo microarray analysis (9). These data reiterate nutraceuticals’ ability to alter epigenetics through nutrient-gene interaction in bone and skeletal homeostasis. **Table 2** represents clinical trials of various bioactive against osteoarthritis.

**Table 2:** Therapeutic effects of curcumin in managing knee osteoarthritis: a collection of clinical interventions.

Bioactives	Sample size, duration & measure	Key outcome	References
Curcumin extract (Curcugen^®^) (500mg/twice/d)	N=101, 8 wk - KOOS - (JOA) -Performance based testing	-Reduced KOOS and JOA score -Improvement in performance-based testing	(208)
Curcumin (500mg/thrice/d)	N=139, 4 wk -KOOS -PGA score	-Efficacy of curcumin was similar to diclofenac -Exhibited better tolerance to side effects of diclofenac	(209)
Curcuminoids (CuraMed^®^) @ 500mg/thrice/d Curcuminoids and Boswellic acid (Curamin^®^) @ 500mg/thrice/d	N=201, 12 wk -WOMAC score -Physical Performance	-CuraMed improved physical performance -Curamin showed significant increase in both physical performance and WOMAC score	(210)
Curcumin (300mg) + Gingerols (7.5mg) + Piperine (3.75mg) Mixture twice/d)	N=60, 2 wk -Serum markers (PGE_2_)	Similar efficacy compared to Naproxen (NSAID) in reducing prostaglandin E_2_ (PGE_2_) in patients	(211)
CartiJoint Forte (Glucosamine hydrochloride, Chondroitin sulfate and Bio-curcumin) (twice/d)	N=53, 12 wk -VAS Score -Lequesne Index score -Physical therapy	Improved VAS score and Lequesne Index Scores when supplemented in addition to physical therapy	(212)
Curcumin extract (bio-optimized)	N=150, 3 mo - PGADA -KOOS Score - Serum parameters	Extract showed decrease in PGADA, KOOS score and reduction in serum levels of sColl2-1.	(213)
Curcumin (100mg/twice/d)	N=107, -VAS score -WOMAC score -Range of Motion (ROM) - LKSS -Serum markers	Curcumin improved inflammation and joint mobility and reduced side effects compared to Ibuprofen (NSAID)	(214)
Curcumin (Sinacurcumin^®^) (80mg/d)	N=30, 3 mo -VAS score -Serum parameters	Decrease in VAS scores, CRP levels, and immunomodulatory effects	(215)
Curcuminoid capsules (500mg/thrice/d) Co-administered with Piperine (15mg/d)	N=40, 6 wk -Serum parameters	Supplementation mitigated systemic oxidative stress measured by SOD, GSH, and MDA Showed anti-inflammatory markers measured by serum IL-4, IL-6, hs-CRP, and TNF-α.	(216) (216)
Curcumagalactomannoside (400mg/d)	N=72, 6 wk -Walking performance -VAS score -WOMAC score	Alleviated symptoms and pain measured by walking performance, VAS and WOMAC scores	(217)
Curcumagalactomannoside (400mg/twice/d) Co-supplemented with Glucosamine hydrochloride (500mg/twice/d)	N=80, 12 wk -VAS score -WOMAC score -KPS score	Improved stiffness, pain and mobility measured by VAS, KPS and WOMAC scores.	(218)
Curcumin (168mg/d) #	N=106, 12 wk Visual Analogue Scale (VAS) score	No significant reduction in VAS score between case and control	(219)
Nanocurcumin (40mg/twice/d)	N=71, 6 wk -WOMAC score	Significant decrease in overall WOMAC score for pain	(134)

# hand osteoarthritis.

mo, Month; wk, Week; d, Day; (CRP), C- reactive Protein; (SOD), Superoxide Dismutase; (GSH), Glutathione; (MDA), Malondialdehyde; (NSAID), Non-Steroidal Anti-inflammatory Drug; (IL), Interleukin; (TNF-α), Tumor Necrosis Factor-α; (VAS), Visual Analog Scale; (JOA), Japanese Orthopedic Association Score for Osteoarthritic knees; (WOMAC), Western Ontario and McMaster Universities Arthritis Index; (PGADA), Patient Global Assessment of Disease Activity; (sColl2-1), Serum Type 2 Collagen; (KPS), Knee Pain Scale; (LKSS), Lysholm Knee Scoring Scale; (KOOS), Knee Injury and Osteoarthritis Outcome Score.

### 4.4 Vitamin D and omega-3 fatty acids

The vitamin D is synthesized beneath the skin upon exposure to ultraviolet-B radiation from the sunlight. This biologically inactive form of vitamin D undergoes a series of reactions, including hydroxylation in the liver and kidney, to convert to an active form such as calcitriol (1,25-dihydroxy vitamin D). Calcitriol increases calcium reabsorption after being filtered by the kidneys. It mobilizes calcium in the bones and maintains calcium and phosphorous homeostasis in bones. Vitamin D deficiency leads to the disease rickets in children due to inadequate bone mineralization, while osteomalacia is reported in adults under similar conditions. Vitamin D deficiency has been associated with elevated inflammation, and long-term vitamin D insufficiency increases the risk of osteoporosis.

The bioactive vitamin D3 is the predominant form found in food. However, the roles of bioactive vitamin D on intestinal homeostasis and its impact on bone metabolism are unknown. Vitamin D and its receptor signaling play an essential role in maintaining intestinal homeostasis and the healthy gut microbiota by modulating effector T cells (Th1 and Th17) and regulatory T cells (220–222). Since vitamin D plays such a crucial role in maintaining gut homeostasis, it is evident that any deficiency will result in altered microbial composition. Several studies recently reported strong links between vitamin D synthesis, exposure to sunlight, and gut microbes, including mice, zebrafish, and humans (223, 224). Supplementation with vitamin D increased *Firmicutes, Bacteroidetes*, and *Actinobacteria* abundance at the phyla level and *Faecalibacterium*, *Ruminococcaceae*, *Coprococcus*, and *Akkermansia* at the genus level. Firmicutes are directly associated with vitamin D serum levels (225, 226). In rats, vitamin D deficiency impaired glucose tolerance and increased D*esulfovibrio, Peptococcus, Roseburia, Lachnopriraceae, Lachnoclostridium*, and *Ruminiclostridium*. *Blautia* linked to 2-picolinic acid was decreased, leading to a decrease in the metabolite that is crucial in generating an immunological response (227).

An association between serum vitamin D, ultraviolet rays (UV-B), and the gut microbiome was studied in the Canadian population. Exposure to narrow band (NB)-UVB among those who did not take vitamin D supplementation led to an aberrant increase in serum vitamin D levels in addition to increasing α- and β-diversity of the gut microbiome. This increase in diversity was similar to the microbial composition of those who took vitamin D supplements, among whom the NB-UVB exposure did not create a difference (228). The gut microbial composition in a semi-nomadic hunter-gatherer (Yanomami) showed similar to those with NB-UVB exposure in the previous study, thus establishing the role of sunlight and vitamin D levels in modulating the gut microbiota (229, 230). The association of dietary omega-3 fatty acids with vitamin D, as proven by several studies (231, 232), establishes the importance of omega-3 and other vital nutrients that directly or indirectly cause variation in the gut microbial composition and thereby affect the progression of inflammatory and musculoskeletal disorders. This aligns with the assumption that nutrition and diet are the significant causatives that trigger various diseases via intestinal dysbiosis.

The essential roles of long-chain fatty acids were evidenced first from the abnormal calcification due to essential fatty acid deficiency (233), suggesting the critical involvement of lipid mediators in bone metabolism. During acute inflammation, the immune cells are regulated by specialized lipid mediators such as lipoxins (derived from arachidonic acid, ARA), resolvins, maresins, and protectins (derived from eicosapentaenoic acid, EPA, and docosahexaenoic acid, DHA), those are collectively known as SPMs (specialized pro-resolving mediators), drive resolution of inflammation, remove inflammatory lesions and promotes apoptotic cell clearance through lymphatics (234). Resolvin E1 (RvE1), derived from EPA (omega-3), and other lipid mediators play a role in inflammation-associated models of arthritis (235) by modulating Cyclooxygenase 2 (COX-2) activity. Data showed that RvE1protectst inflammatory-induced bone damage by regulating bone cell gene expression in a model (236). Phospholipase-mediated releases of ARA, the substrate for prostaglandin E2 (PGE2) synthesis, are also involved in signaling bone turnover. COX2 converts ARA to PGE2 and modulates both osteoclastogenesis by stimulating the expression of RANK, RANKL, and osteoblastogenesis by promoting insulin-like growth factor (IGF-1) and Wnt signalling, respectively (237). PGE2 levels are a potent modulator of resorption and bone formation, while elevated PGE2 suppresses osteoblasts and promotes bone differentiation and resorption by osteoclasts, whereas low levels stimulate bone formation by osteoblasts (238). There is strong evidence that a diet rich in ω-3 fatty acid downregulates markers related to oxidative stress, cartilage degradation, and inflammation in chondrocytes while these markers are elevated with a higher ω-6 fatty acids (7, 239–243). Therefore, the quality of polyunsaturated fatty acids (PUFA) might have a distinct role in bone metabolism since metabolites derived from ω-6 and ω-3 fatty acids can act on precursor cells of osteoblast and adipocytes differentially (244). Bioactive rich in ω-6 fatty acids raises ω-6/ω-3 ratio and triggers adipogenesis lineage of the bone marrow by promoting adipogenic differentiation of mesenchymal cells, diverting their differentiation into osteoblasts and thus disrupting the homeostasis of the bone remodeling.

In contrast, bioactive rich with ω-3 fatty acids do not exert a strong adipogenesis induction and promote an inhibitory effect on osteoclastogenesis that helps maintain the bone mineral mass and thus allows osteoblastogenesis. Omega-3 fatty acids also regulate musculoskeletal growth (245) and bone homeostasis by causing a reduction in bone resorption when bone formation is consistent (238). Studies suggest that omega-3 fatty acids exert their beneficial effects on bone strength by improving microarchitecture with concomitant increases in trabecular network with decreased trabecular space, trabecular number, bone volume fraction and bone surface density (246, 247). Mice fed with fish oil after ovariectomy showed a reduced loss of bone mineral density (248), while femur bone mineral content positively correlated with ω-3 PUFAs in ovariectomized rats (249). DHA has been shown to accumulate in bone periosteum and bone marrow and correlates positively with bone mineral content (250). The ω-3 PUFAs contribute to the growth phase of the developing skeleton by accelerating growth plate, bone growth, chondrocyte proliferation and differentiation that collectively results in superior trabecular and cortical structure to stiffer bones and improved bone quality as evidenced in *fat-1* mice (7). Clinical studies reiterated that ω-3 PUFAs may be linked with reduced structural damage in bones and improved function and pain in knee and hip OA patients (8, 251, 252).

A Rancho Bernardo study emphasized the roles of α-linoleic acid (ALA), EPA, and DHA in bone health to understand the relationship between ω-6/ω-3 ratio and bone mineral density (253). A higher percentage was associated with less bone mineral density (BMD), suggesting the crucial role of dietary ω-3 PUFA in preventing bone ailments. A similar study was conducted in older women and concluded dietary PUFA positively modulates BMD in the lumbar spine (L2-L4) as well as BMD of the total body (254). Studies have shown that ω-3 PUFAs and metabolites influence bone remodeling (255). The role of omega-3 fatty acids as a nutraceutical in managing osteoarthritis revealed that both oxidative stress and inflammation are involved (256). The ω-3 PUFAs and their metabolites have anti-inflammatory, antioxidant, and analgesic properties, which prevent bone loss by upregulating the Akt pathway and inhibiting the iNOS pathway (255), NF-κB pathway, MAPK signaling, JNK pathway, IL-1β, COX-2, and MMP-13. The pathogenesis of osteoarthritis has also been linked to adiposity. Adiposity is a potent risk factor for bone ailments with increased leptin levels. While excess ω-6 PUFAs are known to cause inflammation during adiposity, ω-3 PUFAs overturn this, reducing the risk and preventing bone ailments. The imbalance of the optimal ω-6/ω-3 ratio has also been linked to osteoarthritis. Hence, the use of ω-3 fatty acids, particularly EPA or DHA, as a nutraceutical supplementation resolves the skewed ω-6/ω-3 ratio, helps reduce inflammation, and results in the management of osteoarthritis and other bone-related conditions (257). High levels of ω-6 PUFA also cause synovial membrane inflammation due to their ability to produce pro-inflammatory eicosanoids (258). Available data suggest that bioactive rich with ω-3 PUFAs could have multiple benefits in modulating bone turnover, including its formation, re-absorption, and density of the bone cells and as an adjuvant with anti-inflammatory drugs to lower the systemic inflammation associated with OA.

Alterations in the ω-6/ω-3 ratio also affect the gut microbiome. Consumption of high ω-6 fatty acids led to low-grade inflammation at the systemic level, whereas ω-3 fatty acids increased the production of intestinal alkaline phosphatase that modulates gut homeostasis (259). Marine molluscs like *Perna canaliculus* contain high levels of omega-3 and various carbohydrates and proteins. Clinical studies have shown that mussel extract has anti-inflammatory effects and significantly affects the intestinal microbiome by reducing the abundance of *Clostridia* species (260). Fish oil rich in DHA, upon supplementation in ob/ob mice, results in significant production of SCFA, reducing the cholesteryl esters and triglycerides. DHA was found to upend intestinal dysbiosis and inhibit lipid droplet formation in the liver. The abundance of *Akkkermansia* and *Muribaculaceae* increased, whereas a reduction was observed in *Lachnospiraceae*, a pathogenic bacterium (261). Clinical interventions with various bioactive supplemented in knee osteoarthritis patient and its outcome are captured in **Table 1**.

## 5 Conclusions

Systemic inflammation is the root cause of most musculoskeletal disorders, where gut dysbiosis and pro-inflammatory triggers strongly modulate disease progression. The interplay between the gut, immune system, and local tissue metabolism drives osteoarthritis pathophysiology. Intestinal dysbiosis disrupts the abundance of essential commensals that protect the mucosal integrity of gut permeability, maintain immune tolerance, produce key metabolites or vitamin synthesis, and generate immune responses against pathogenic bacteria. Moreover, inflammatory states in osteoarthritis change microflora and accumulate bacterial LPS. TLR4 receptor activation is critical in regulating gut permeability and is associated with acute and chronic inflammation. The dysregulated TLR signaling negatively impacts the gut, compromising its permeability and resulting in inflammation and dysbiosis. Modulators of TLR signalling can play a crucial role in maintaining gut homeostasis. Despite the evidence, the specific sets of gut microbiotas in OA still needs to be understood in detail. Most of the evidence on the role of gut microbiota in OA, obtained from animal models, thus warrants control clinical trials. The association of dietary omega-3 fatty acids with vitamin D directly or indirectly causes variation in the gut microbial composition, affecting the progression of inflammatory and musculoskeletal disorders. The intestinal microbiome’s various roles in modulating the immune system and their impact by Bioactives still need to be understood. Further, the role of gut microbiome has yet to be elucidated in the various clinical trials, for example, bioactive curcumin used as an intervention against osteoarthritis (**Table 2**).

Curcumin promised to revert LPS-induced inflammation by improving synovial endotoxemia and releasing a specific set of gut microbiotas that potentially improve osteoarthritis functions. The absence of curcumin detected in the subjects’ serum after large intake indicates that anti-oxidant, anti-inflammatory, and other beneficial effects reported by several studies could mediate by signaling receptor activation. Curcumin might target NLRP3 inflammasome by reducing NLRP3-mediated inflammation and oxidative stress and possibly ameliorating osteoarthritis pathophysiology. Curcumin has the same vanilloid ring pharmacophore in its structure as capsaicin, which rationalizes curcumin’s affinity for the activation of TRPV1. Thus, the potential mechanisms involving nociception in pain-modulating pathways via TRV1 channel signaling are emerging with these Bioactives.

Bioactives could be potential alternative therapeutic in managing chronic inflammatory diseases like osteoarthritis. However, various confounders like geography, dietary habits, exposure to sunlight, serum vitamin D levels, history of medication, infection, endogenous gut microbial diversity, and other variability at inter-individual and intra-individual levels could influence the beneficial effects of Bioactives in managing osteoarthritis pathophysiology. The article highlighted the importance of bioactive compounds in preventing and treating OA. However, further work is required involving advanced technology to separate more bioactive compounds and explore the exact molecular mechanism and therapeutic targets of the bioactive compounds, which will be helpful for the treatment of OA in the early stage, preventing disability and enhancing the quality of patients’ lives.

## Author contributions

SB: Conceptualization, Writing – original draft, Writing – review & editing. KS: Writing – original draft. AD: Conceptualization, Writing – original draft, Writing – review & editing.
